# Investigating the molecular mechanism of positive and negative allosteric modulators in the calcium-sensing receptor dimer

**DOI:** 10.1038/srep46355

**Published:** 2017-04-18

**Authors:** Stine Engesgaard Jacobsen, Ulrik Gether, Hans Bräuner-Osborne

**Affiliations:** 1 Department of Drug Design and Pharmacology, Faculty of Health and Medical Sciences, University of Copenhagen, Denmark; 2 Molecular Neuropharmacology and Genetics Laboratory, Lundbeck Foundation Center for Biomembranes in Nanomedicine, Department of Neuroscience and Pharmacology, Faculty of Health and Medical Sciences, University of Copenhagen, Denmark

## Abstract

Allosteric modulators that are targeting the calcium-sensing receptor (CaSR) hold great therapeutic potential, and elucidating the molecular basis for modulation would thus benefit the development of novel therapeutics. In the present study, we aimed at investigating the mechanism of allosteric modulation in CaSR by testing dimers carrying mutations in the allosteric site of one or both of the subunits. To ensure measurements on a well-defined dimer composition, we applied a *trans*-activation system in which only the specific heterodimer of two loss-of-function mutants responded to agonist. Although one of these mutants was potentiated by a positive allosteric modulator, we showed that receptor activity was further potentiated in a *trans*-activation heterodimer containing a single allosteric site, however only when the allosteric site was located in the subunit responsible for G protein coupling. On the contrary, preventing activation in both subunits was necessary for obtaining full inhibition by a negative allosteric modulator. These findings correlate with the proposed activation mechanism of the metabotropic glutamate receptors (mGluRs), in which only a single transmembrane domain is activated at a time. CaSR and mGluRs belong to the class C G protein-coupled receptors, and our findings thus suggest that the activation mechanism is common to this subfamily.

The calcium-sensing receptor (CaSR) is essential for the maintenance of calcium homeostasis as it continually monitors the extracellular level of calcium^1^. The receptor is expressed at high levels in the parathyroid glands, thyroid glands, kidneys and bones where its signaling controls secretion of calcium-elevating and –decreasing hormones as well as absorption and excretion of calcium^2^. CaSR is moreover one of the few G protein-coupled receptors (GPCRs) in which a large number of naturally occurring mutations has been identified^3^,^4^. Importantly, many of these have been directly linked with severe diseases thus emphasizing the pathophysiological importance of CaSR^5^.

In GPCR drug discovery it can be advantageous to focus on the allosteric binding site, as the orthosteric site is highly conserved thereby making it difficult to achieve receptor selectivity for orthosteric ligands. Furthermore, allosteric drug compounds might be less likely to show adverse effects, since the modulatory effect is dependent on the presence of agonist^6^. Cinacalcet is a positive allosteric modulator (PAM) targeting CaSR and the very first GPCR allosteric modulator to get regulatory approval. It is used to treat secondary hyperparathyroidism in end-stage renal disease^7^, primary hyperparathyroidism where patients are unable to undergo parathyroidectomy^8^ and severe hypercalcemia in patients with parathyroid carcinoma^9^. The use of Cinacalcet is however limited due to severe adverse effects^10^, and improved allosteric drug compounds are thus in request.

CaSR belongs to the class C GPCRs and contains the structural features that are characteristic for this receptor subfamily including a large amino-terminal domain (ATD) containing the orthosteric binding sites^11^,^12^,^13^,^14^ as well as the seven transmembrane (7TM) domain that is common to all GPCRs. Class C receptors function either as homo- or heterodimers at the cell surface, and homodimerization has indeed been verified for CaSR^15^,^16^,^17^,^18^. Most mechanistic studies of the class C receptors have been conducted on the metabotropic glutamate receptors (mGluRs), and only limited information about the mode of action in CaSR is currently available. For the mGluRs, agonist binding in the ATDs has been shown to trigger conformational changes from an open to a closed state resulting in a change in the relative orientation of the two ATDs in the dimer^19^,^20^,^21^. This triggers further rearrangements at the 7TM dimer interface, which is involved in mediating activation and subsequent G protein coupling in the 7TMs^22^,^23^,^24^,^25^. In the heterodimeric γ-amino butyric acid type B (GABA_B_) receptors, the GABA_B1_ subunit is responsible for agonist binding while G protein coupling occurs only in the GABA_B2_ subunit^26^,^27^,^28^, and interestingly, it has also been reported that only one 7TM domain at a time is activated in the mGluR dimer^29^,^30^. Collectively, this demonstrates an asymmetry in the activation mechanism of class C receptors despite the requirement for dimerization. While recently published crystal structures of the ATD in CaSR confirmed that this receptor undergoes domain closure with formation of a dimer interface that is critical for activation of the receptor^13^,^14^, the 7TM domains in which the allosteric site is located has yet to be studied. Fully elucidating the mechanism of action and modulation in the 7TM domains could facilitate the development of novel allosteric drug compounds of CaSR.

In this study, we aimed to investigate the role of allosteric modulation in the CaSR dimer. Specifically, we examined whether it is a necessity to have modulators bound in both subunits or if one modulator per dimer is adequate. Our data suggest that a single allosteric site per CaSR dimer is sufficient for obtaining positive modulation of activity, while full inhibition is only achieved if activation of both 7TM domains is prevented.

## Results

### CaSR is able to signal via *trans*-activation of the dimer

When studying receptor dimers, it is of crucial importance to ensure that functional readouts arise only from a single, well-defined subunit composition. Here, we applied a *trans*-activation assay, which has previously been demonstrated for the mGluRs^31^,^32^ and CaSR^33^,^34^. Upon co-expression of a mutant preventing agonist activation and a mutant preventing G protein coupling, a recovery of functional response can be observed although each of the mutants is non-functional on its own. This functional response is specifically mediated by a heterodimer composed of the two mutants, as ligand binding occurring in the subunit with impaired G protein coupling triggers G protein coupling in the subunit with impaired ligand binding. The *trans*-activation mechanism thus allows for an assay in which a measured functional response can only arise from one specific dimer. Accordingly, two types of inactivating CaSR mutants were required in the present study in order to utilize the *trans*-activation system.

**Figure 1:**
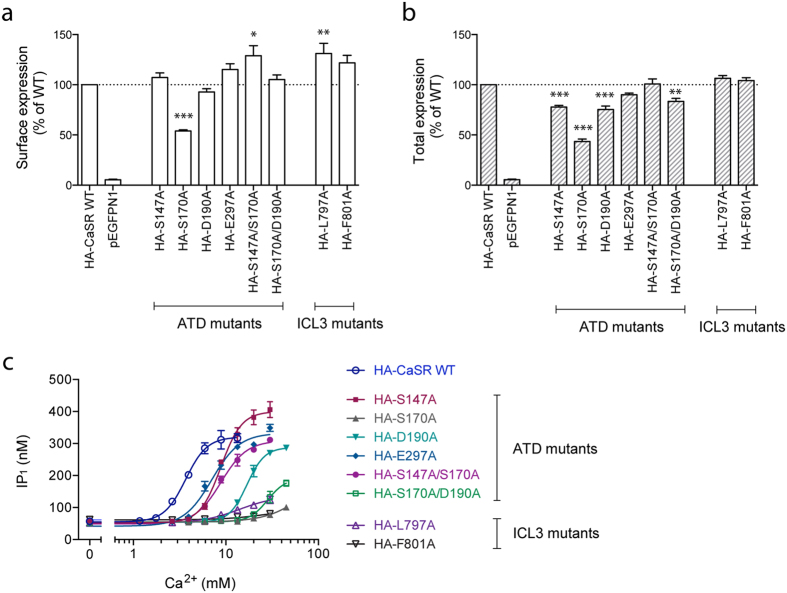
Expression levels and functional characterization of mutations located in the amino-terminal domain (ATD) or intracellular loop 3 (ICL3) of CaSR. HEK293T cells transiently transfected with the different HA-tagged CaSR mutants were tested. The (**a**) surface and (**b**) total expression levels of the CaSR mutants were determined by using anti-HA antibodies in ELISA. Data are shown as percentage of the expression level of WT HA-CaSR and are means ± S.E.M. of three independent experiments performed in triplicates. Statistical analysis was performed using one-way ANOVA followed by Dunnett’s post-test in which each mutant was compared to the WT receptor (*P < 0.05, **P < 0.01, ***P < 0.001). (**c**) The function of HA-CaSR WT and mutants was assessed using the IP-One assay, which measures increases in d-myo-inositol monophosphate (IP_1_) upon activation of the G_q_ signaling pathway. The endogenous agonist Ca^2+^ was tested in increasing concentrations. Data are means ± S.D. of a single representative experiment performed in triplicates. Two additional experiments gave similar results.

For class C receptors, a highly conserved orthosteric binding site is found in the cleft between the two lobes of the ATD^35^. Based on previously published mutagenesis studies of CaSR^11^,^34^,^36^, residues in this binding site were chosen for mutation. G protein coupling is particularly dependent on conserved residues in the intracellular loop 3 (ICL3) of class C GPCRs^27^,^37^,^38^,^39^, hence residues in this part of the receptor were also chosen for mutation. All mutant constructs were tagged with an HA epitope allowing for validation of surface and total expression by use of an enzyme-linked immunosorbent assay (ELISA). Data showed that all mutants were expressed at the surface to the same extent or in significant higher levels than HA-tagged WT CaSR, except the S170A mutation, which only displayed 54% surface expression and 43% total expression compared to WT (Fig. 1a,b). S147A, D190A and S170A/D190A displayed a minor, yet significant, decrease in total expression (Fig. 1b). Functional characterization of the mutants was conducted by testing the endogenous agonist Ca^2+^ in the IP-One assay, which measures activation of the G_q_ signaling pathway. None of the six constructs with mutations in the conserved orthosteric binding site displayed complete loss of function, however lower potency of Ca^2+^ was observed for all of them (Fig. 1c and Table 1). Only the S170A mutant displayed severe functional impairment with highly reduced potency and max response of only 17% of the WT response at the highest tested Ca^2+^ concentration. The two constructs with mutated ICL3 regions likewise demonstrated highly reduced potency of Ca^2+^ and only 25% (for L797A) and 5.9% (for F801A) of the WT maximum response (Fig. 1c and Table 1).

In spite of lower expression levels, S170A was chosen for further studies, as this mutation resulted in the greatest loss of function compared to the other mutations in the orthosteric binding site. Henceforward, the S170A construct will also be referred to as the ATD mutant. Of the two constructs with mutations in ICL3, F801A was chosen as it demonstrated complete loss of function while no impairment of receptor expression was observed. F801A will be referred to as the ICL3 mutant.

**Figure 2:**
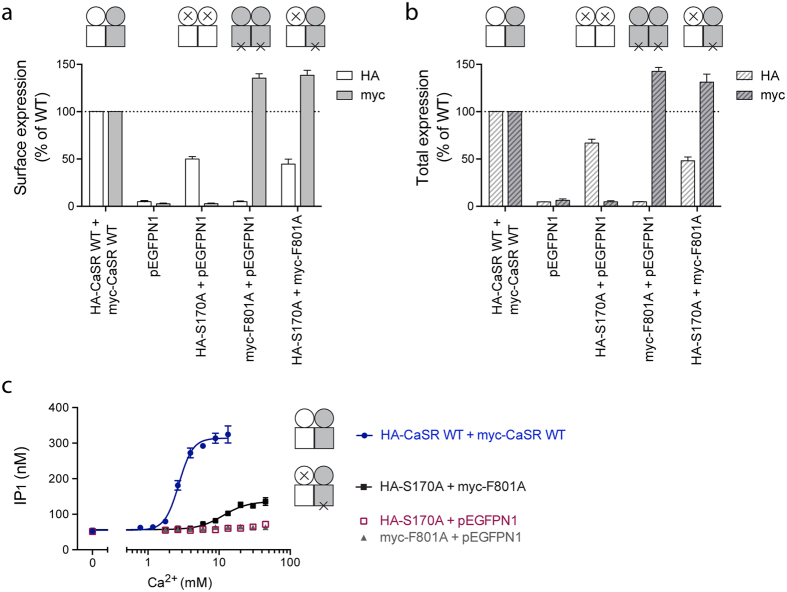
*Trans*-activation of the CaSR heterodimer. HEK293T cells transiently transfected with a HA-tagged CaSR construct (or pEGFPN1) and a myc-tagged CaSR construct (or pEGFPN1) in a 1:1 ratio were tested. The (**a**) surface and (**b**) total expression levels of the CaSR constructs were determined by using ELISA and either anti-HA antibodies or anti-myc antibodies as indicated in the figure. Data from using anti-HA antibody are shown as percentage of the expression level of WT HA-CaSR while data from using anti-myc antibody are shown as percentage of the expression level of WT myc-CaSR. All data are means ± S.E.M. of three independent experiments performed in triplicates. (**c**) Functional characterization was performed using the IP-One assay, which measures increases in d-myo-inositol monophosphate (IP_1_) upon activation of the G_q_ signaling pathway. Ca^2+^ was tested in increasing concentrations. Data are means ± S.D. of a single representative experiment performed in triplicates. Two additional experiments gave similar results.

These two mutants were subsequently investigated for heterodimeric *trans*-activation. In order to confirm expression of both mutants upon co-transfection, the HA-tag in the F801A construct was replaced with a myc-tag. In accordance with results described above, ELISA experiments demonstrated lower expression of HA-CaSR-S170A compared to WT HA-CaSR, while myc-CaSR-F801A displayed increased expression compared to WT myc-CaSR. Importantly, the expression levels of each mutant were similar whether the mutant was co-expressed with the pEGFPN1 vector or co-expressed in the heterodimer (Fig. 2a,b), and co-transfection of the ATD and ICL3 mutants did consequently not influence the expression levels of each mutant. When tested in the functional IP-One assay, myc-CaSR-F801A was shown to be non-functional at concentrations up to 45 mM Ca^2+^, whilst HA-CaSR-S170A only displayed a very small increase in receptor activity at this concentration (Fig. 2c). Upon co-expression of the two mutants, a functional response constituting 30% of the WT CaSR response was detected (Fig. 2c, Table 2), which must arise from signaling in the S170A:F801A heterodimer. The potency of Ca^2+^ was decreased for the *trans*-activation response as observed from a rightward shift in the concentration-response curves (Fig. 2c) and the increase in EC_50_ of Ca^2+^ from 2.98 mM for WT to 12.7 mM for the heterodimer response (Table 2). In conclusion, the functionally impaired S170A and F801A mutants successfully demonstrated recovery of activity upon co-expression, hence validating trans-activation of the CaSR dimer. In the present study, this trans-activation assay was thus used to ensure measurements on dimers with specific subunit compositions when investigating allosteric modulation in the CaSR dimer.

### The mutation E837A eliminates effects from both positive and negative allosteric modulators

**Figure 3:**
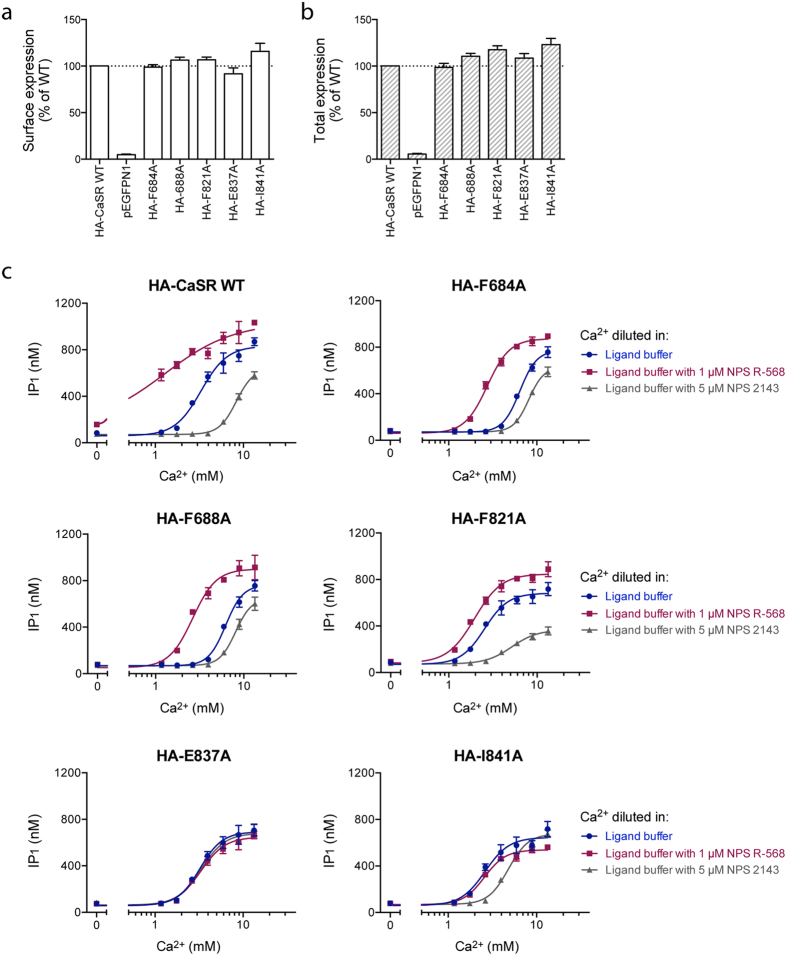
Expression levels and functional characterization of mutations located in the allosteric site of CaSR. HEK293T cells transiently transfected with the HA-tagged CaSR constructs were tested. The (**a**) surface and (**b**) total expression levels of the CaSR mutants were determined by using anti-HA antibodies in ELISA. Data are shown as percentage of the expression level of WT HA-CaSR and are means ± S.E.M. of three independent experiments performed in triplicates. (**c**) The function of HA-CaSR WT and mutants was assessed using the IP-One assay, which measures increases in d-myo-inositol monophosphate (IP_1_) upon activation of the G_q_ signaling pathway. The endogenous agonist Ca^2+^ was tested in increasing concentrations in the presence and absence of 1 μM NPS R-568 (positive allosteric modulator) and 5 μM NPS 2143 (negative allosteric modulator), respectively. Data are means ± S.D. of a single representative experiment performed in triplicates. Two additional experiments gave similar results.

Binding sites for positive allosteric modulators (PAMs) and negative allosteric modulators (NAMs), respectively, have been identified in the 7TM region of CaSR. As these sites are overlapping although not identical, it should be possible to identify a mutation impairing effects of PAMs and NAMs. Based on mutagenesis studies reported in the literature^40^,^41^,^42^,^43^, five single point mutations in the allosteric site were generated, and ELISA experiments demonstrated that the constructs were all expressed to the same extent as WT HA-CaSR (Fig. 3a,b). Functional characterization was performed by testing increasing concentrations of Ca^2+^ in the presence or absence of the PAM NPS R-568 or the NAM NPS 2143. All mutations except E837A affected the potency of Ca^2+^ as they demonstrated a significant increase (F684A and F688A) or decrease (F821A and I841A) in the EC_50_ of Ca^2+^ compared to the WT receptor (Fig. 3c, Table 3), and these mutations thus interfered with the agonist-mediated signaling of the receptor although being located in the allosteric site. For the WT receptor, as well as the mutants F684A and F688A, EC_50_ values of Ca^2+^ were significantly decreased upon addition of PAM, while the presence of a NAM significantly increased the EC_50_ of Ca^2+^ for the WT receptor and all mutants except E837A (Fig. 3c, Table 3). For the E837A mutant, neither NPS R-568 nor NPS 2143 had any significant effect on the Ca^2+^-induced response (Fig. 3c, Table 3), and this mutation thus successfully impaired the overlapping PAM and NAM sites. For that reason, E837A was chosen for further studies.

### Co-expression of an ATD and ICL3 mutant allows for surface expression of specific ATD:ICL3 heterodimers

**Figure 4:**
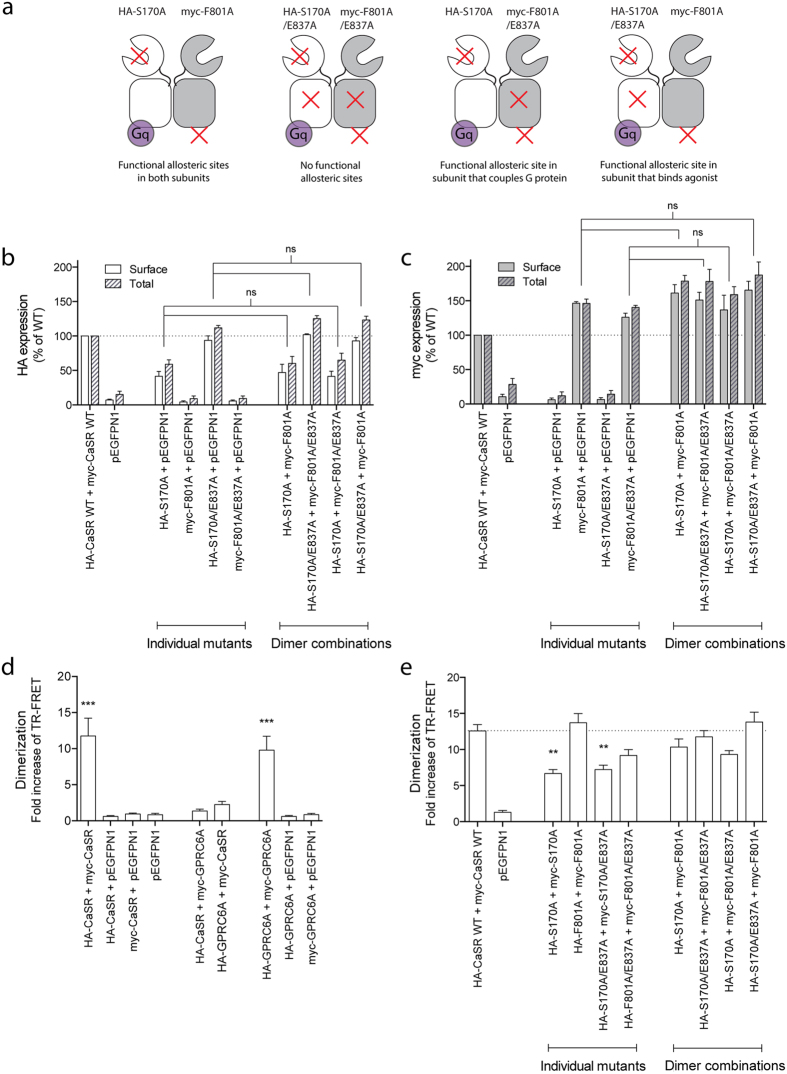
Validation of the expression levels and dimerization of the four CaSR heterodimers of interest. HEK293T cells transiently transfected with a HA-tagged construct (or pEGFPN1) and a myc-tagged construct (or pEGFPN1) in a 1:1 ratio were tested. (**a**) Schematic figure depicting the four heterodimers of interest. Each dimer contains the ATD mutant in which activation by agonist is prevented and the ICL3 mutant in which G protein coupling is prevented. As a consequence, only the heterodimer is functional active. (**b**) and (**c**) Surface and total expression were assessed for each mutant upon co-expression with pEGFPN1 or another mutant. Expression levels were determined using ELISA and (**b**) anti-HA antibodies or (**c**) anti-myc antibodies. Data from measurements of surface expression are shown as percentage of the surface expression of WT CaSR, while measurements of total expression are shown as percentage of the total expression of WT CaSR. All data are means ± S.E.M. of three independent experiments performed in triplicates. Statistical analysis was performed using one-way ANOVA followed by Tukey post-test (ns, *P* > 0.05). (**d**) and (**e**) Dimerization of HA-tagged receptor constructs and myc-tagged receptor constructs was assessed using the TR-FRET dimerization assay, which measures FRET between a donor fluorophore conjugated to an anti-HA antibody and an acceptor fluorophore conjugated to an anti-myc antibody. Constructs were co-expressed and tested as indicated in the figure. (**d**) WT receptor constructs were tested for validation of the assay. (**e**) Mutant CaSR constructs were tested as homodimers and upon co-expression in the four heterodimers of interest. The FRET ratio (acceptor emission/donor emission) for each condition was normalized to the FRET ratio of pEGFPN1. Data are means ± S.E.M. of three independent experiments performed in triplicates. Statistical analysis was performed using one-way ANOVA followed by Dunnett’s post-test in which each condition was compared to (**d**) pEGFPN1 or (**e**) WT CaSR (**P < 0.01, ***P < 0.001).

Because the E837A mutation eliminates the allosteric modulation in the subunit that contains the mutation, it was possible to identify whether the functional allosteric site in the other subunit was sufficient for obtaining the modulatory effect in the dimer. Four specific dimers were tested and the *trans*-activation control system was used to ensure measurements on the specific subunit composition in each; a dimer without any mutations in the allosteric sites, a dimer with an allosteric site mutation in one subunit, a dimer with a allosteric site mutation in the other subunit and a dimer with mutated allosteric sites in both subunits. The four dimers of interest are shown schematically in Fig. 4a. For all of them, each subunit in itself should be nonfunctional due to the ATD or ICL3 mutant, and although the mutant homodimers would also be present at the cell surface, the only active dimer composition would thus be the heterodimer of interest.

At first, receptor expression was validated for each mutant upon co-expression in the four dimer combinations of interest. In accordance with results described above, the S170A mutant displayed only 42% surface expression and 59% total expression compared to WT HA-CaSR upon co-expression with pEGFPN1. When introducing the allosteric site mutation E837A in the HA-S170A construct, expression levels increased to the level of WT HA-CaSR expression (Fig. 4b). Both myc-F801A and myc-F801A/E837A were expressed in levels corresponding to about 140% of the WT myc-tagged receptor (Fig. 4c). When co-transfecting mutants in the four dimer combinations, the expression level of each mutant was not significantly different from the expression level upon co-expression with pEGFPN1 (Fig. 4b,c), and the heterodimer co-expression did consequently not interfere with the expression levels of each subunit.

Dimerization between a myc-tagged receptor construct and a HA-tagged receptor construct can be validated by use of a time-resolved fluorescence resonance energy transfer (TR-FRET) dimerization assay^44^. When adding an anti-HA antibody conjugated to a donor fluorophore and an anti-myc antibody conjugated to an acceptor fluorophore, FRET occurs when the two fluorophores are in close proximity. This would be the case upon receptor dimerization. At first, the assay setup was validated by co-expressing WT HA-CaSR and WT myc-CaSR, which resulted in an 11.8 fold increase in FRET signal thus confirming homodimer formation. When each of the WT constructs was instead co-expressed with pEGFPN1, no FRET signal could be detected (Fig. 4d). In order to confirm the specificity of the assay, CaSR was tested in the presence of the closest related mammalian receptor GPRC6A. Co-expressing WT HA-CaSR and WT myc-GPRC6A or WT myc-CaSR and WT HA-GPRC6A only triggered very minor, non-significant FRET signals, which were likely to arise merely from the over-expression of both receptors. In comparison, co-expressing WT HA-GPRC6A and WT myc-GPRC6A gave rise to a 9.80 fold increase in FRET signal (Fig. 4d) thereby confirming the previously reported homodimerization of GPRC6A^44^. In conclusion, only specific dimer formation gave rise to an increase in FRET ratio.

Upon co-expressing a myc-tagged and a HA-tagged version of each of the four CaSR mutants, increases in FRET signal were detected for each and homodimerization was thus verified (Fig. 4e). Although the S170A mutant displayed a significant decrease in FRET signal compared to the WT receptor while the FRET signal of the F801A mutant was equal to, if not higher than, the WT receptor (Fig. 4e), these findings correlate with the expression levels determined for each mutant. On the contrary, the S170A/E837A mutant and the F801A/E837A mutant were both expressed in equal or higher levels than the WT receptor, while the FRET signal was decreased compared to WT, although not significantly for F801A/E837A. When testing the four dimer combinations of interest, a large increase in FRET signal was detected for all of them, which confirmed heterodimerization. The increases in FRET signal were not significantly different from that of the WT receptor, and in conclusion, the four heterodimers were present in the cell membrane in levels that were equal to the amount of WT CaSR dimers, although varying expression levels were observed for each individual subunit.

### One allosteric site is sufficient for obtaining potentiation of CaSR

**Figure 5:**
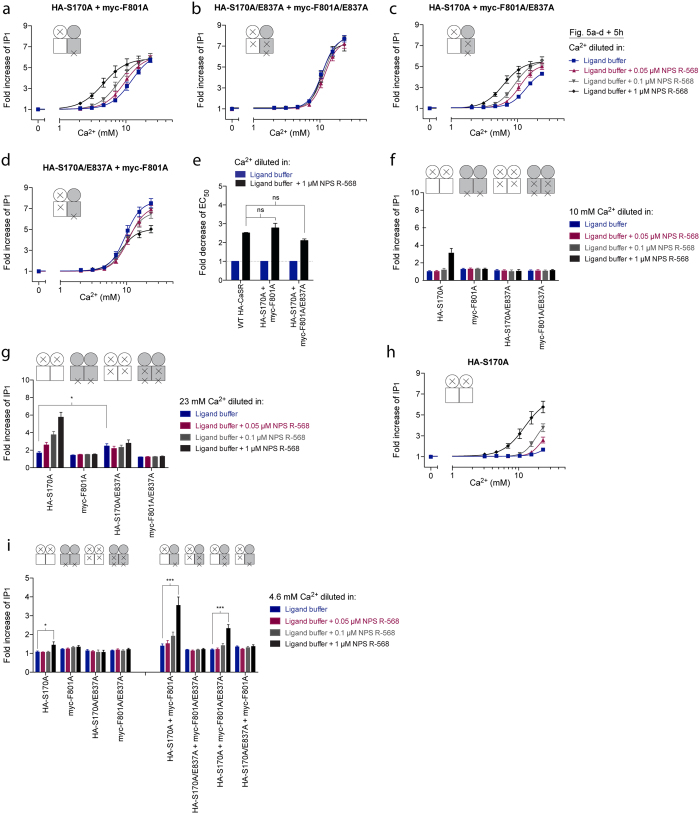
Positive allosteric modulation of the four CaSR heterodimers of interest. HEK293T cells transiently transfected with a HA-tagged CaSR construct (or pEGFPN1) and a myc-tagged CaSR construct (or pEGFPN1) in a 1:1 ratio were tested. Specifically, cells co-expressing (**a**) HA-S170A and myc-F801A, (**b**) HA-S170A/E837A and myc-F801A/E837A, (**c**) HA-S170A and myc-F801A/E837A and (**d**) HA-S170A/E837A and myc-F801A were tested. The function of these heterodimers was assessed using the IP-One assay, which measures increases in d-myo-inositol monophosphate (IP_1_) upon activation of the G_q_ signaling pathway. The endogenous agonist Ca^2+^ was tested in increasing concentrations in the presence and absence of 0.05 μM, 0.1 μM and 1 μM NPS R-568, respectively. (**e**) Effect of 1 μM NPS R-568 on the EC_50_ of Ca^2+^ on WT HA-CaSR and the S170A:F801A and S170A:F801A/E837A heterodimers. EC_50_ values are shown as fold decrease of the EC_50_ value of Ca^2+^ alone for each of the constructs. EC_50_: the ligand concentration that is required for eliciting 50% of the maximum response. Data are means ± S.E.M. of three independent experiments performed in triplicates. Statistical analysis was performed using one-way ANOVA followed by Dunnett’s post-test (ns, *P* > 0.05). (**f**,**g**) The function of each mutant upon co-expression with pEGFPN1 was assessed by testing (**f**) 10 mM Ca^2+^ or (**g**) 23 mM Ca^2+^ in the presence and absence of 0.05 μM, 0.1 μM and 1 μM NPS R-568, respectively. In (**g**), statistical analysis was performed using one-way ANOVA followed by Dunnett’s post-test in which the Ca^2+^ response for each mutant was compared to the Ca^2+^ response of S170A (*P  < 0.05) (**h**) Ca^2+^ concentration-response curves of HA-S170A were generated in the presence and absence of 0.05 μM, 0.1 μM and 1 μM NPS R-568, respectively. Data obtained in Fig. 5a–d,h from stimulation with 4.6 mM Ca^2+^ is depicted in (**i**). Statistical analysis was performed using one-way ANOVA followed by Dunnett’s post-test in which responses in the presence of PAM were compared to the Ca^2+^-mediated response alone for each mutant homodimer or heterodimer (*P  < 0.05, ***P < 0.001). All data are shown as fold increase over the basal level of IP_1_ upon incubation in ligand buffer and are means ± S.E.M. of three independent experiments performed in triplicates.

Potentiation of Ca^2+^-mediated responses from the four heterodimers of interest was investigated by testing increasing concentrations of Ca^2+^ in the presence of 0.05 μM, 0.1 μM and 1 μM NPS R-568, respectively. The S170A:F801A heterodimer contains functional allosteric sites in both subunits and accordingly, leftward shifts of the Ca^2+^ curves were observed thus demonstrating a concentration-dependent potentiation by NPS R-568 (Fig. 5a). On the contrary, the response of the S170A/E837A:F801A/E837A heterodimer was not potentiated, as the heterodimer contains mutations in both allosteric sites, (Fig. 5b). The S170A:F801A/E837A and S170A/E837A:F801A dimers both contain only one functional allosteric site per dimer, however responses of the former was potentiated (Fig. 5c) whilst those of the latter was not (Fig. 5d). In the S170A:F801A/E837A heterodimer, the functional allosteric site is located in the subunit that is responsible for G protein coupling, thus it seems that potentiation can only occur when the functional allosteric site and the G protein coupling site are in the same subunit. In the S170A/E837A:F801A heterodimer, the allosteric site is located in the subunit in which G protein coupling is prevented and accordingly, no potentiation was observed. The Ca^2+^-mediated response of this heterodimer was in fact inhibited by the highest tested concentration of NPS R-568. Importantly, potentiation of the Ca^2+^-induced response in the WT CaSR dimer was not significantly increased compared to the potentiation obtained in the S170A:F801A heterodimer containing two allosteric sites and in the S170A:F801A/E837A heterodimer containing only one site (Fig. 5e), and one allosteric site per dimer thus seemed sufficient for fully potentiating the agonist-mediated response of CaSR.

Although the activity of each subunit in the four heterodimers was impaired by the S170A or F801A mutation, they would nevertheless be present at the cell surface as homodimers. In order to confirm the loss of activity for these homodimers, 10 mM Ca^2+^ was tested at each mutant in the presence or absence of 0.05 μM, 0.1 μM and 1 μM NPS R-568, respectively (Fig. 5f). While F801A, S170A/E837A and F801A/E837A were all inactive upon addition of Ca^2+^ and PAM, an increase in IP_1_ was observed for the S170A construct upon addition of 10 mM Ca^2+^ +1 μM of NPS R-568 although no response was detected for Ca^2+^ alone (Fig. 5f). To study the potential influence from the mutant homodimers at increased agonist concentrations, the highest tested Ca^2+^ concentration from the heterodimer experiments (23 mM Ca^2+^) was likewise tested at each mutant in the presence of 0.05 μM, 0.1 μM and 1 μM NPS R-568, respectively (Fig. 5g). At this Ca^2+^ concentration, F801A and F801A/E837A were still inactive upon addition of Ca^2+^ and PAM, while the S170A/E837A construct elicited a small, yet significant, increase in Ca^2+^-mediated signaling, although potentiation of this agonist response was successfully impaired by the E837A mutation (Fig. 5g). In conclusion, neither of these mutants should thus influence the measurements of potentiation in the heterodimers. Surprisingly, a concentration-dependent potentiation was observed for the S170A construct in spite of the very limited Ca^2+^-mediated activity (Fig. 5g), and this potentiation was thus likely to contribute when measuring on the S170A:F801A and S170A:F801A/E837A heterodimers, as they both contain the S170A subunit. Importantly, the potentiated concentration-response curves of the S170A mutant (Fig. 5h) were right-shifted in comparison to the curves obtained when co-expressing S170A and F801A (Fig. 5a) or S170A and F801A/E837A (Fig. 5c), and the NPS R-568-mediated activity in Fig. 5a and c can therefore not be solely mediated by S170A homodimers. As a consequence, the heterodimers must be partly responsible for the measured potentiation.

To further emphasize these findings, a low Ca^2+^ concentration (4.6 mM), at which the contribution from the S170A homodimer was highly limited, was chosen from the curves shown in Fig. 5a–d,h. For S170A, a very small, yet significant, increase in IP_1_ was observed only at the highest tested PAM concentration, whereas no significant agonist effect or potentiation was observed for any of the other mutants. On the contrary, robust and highly significant potentiation was detected for the responses of the S170A:F801A and S170A:F801A/E837A heterodimers (Fig. 5h). In conclusion, S170A homodimers are likely to contribute when measuring on the S170A:F801A and S170A:F801A/E837A heterodimers, however the S170A homodimers alone can not account for the observed activity, and the heterodimers must thus also be subjected to NPS R-568-mediated potentiation.

### Preventing activation in both subunits of CaSR is required for full inhibition of activity

**Figure 6:**
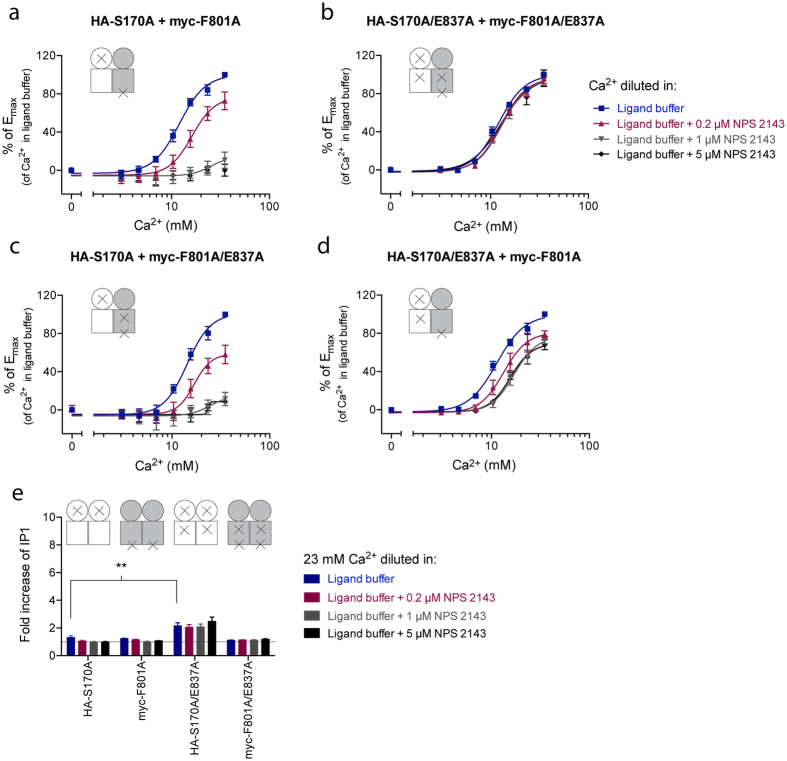
Negative allosteric modulation of the four CaSR heterodimers of interest. HEK293T cells transiently transfected with a HA-tagged CaSR construct (or pEGFPN1) and a myc-tagged CaSR construct (or pEGFPN1) in a 1:1 ratio were tested. Specifically, cells co-expressing (**a**) HA-S170A and myc-F801A, (**b**) HA-S170A/E837A and myc-F801A/E837A, (**c**) HA-S170A and myc-F801A/E837A and (**d**) HA-S170A/E837A and myc-F801A were tested. The function of the heterodimers was assessed using the IP-One assay, which measures increases in d-myo-inositol monophosphate (IP_1_) upon activation of the G_q_ signaling pathway. The endogenous agonist Ca^2+^ was tested in increasing concentrations in the presence and absence of 0.2 μM, 1 μM and 5 μM NPS 2143, respectively. All data are shown as percentages of the maximum response for Ca^2+^ in the absence of NPS 2143 and are means ± S.E.M. of three independent experiments performed in triplicates (**e**) The function of each mutant upon co-expression with pEGFPN1 was assessed by testing 23 mM Ca^2+^ in the presence and absence of 0.2 μM, 1 μM and 5 μM NPS 2143, respectively. Statistical analysis was performed using one-way ANOVA followed by Dunnett’s post-test in which the Ca^2+^ response for each mutant was compared to the Ca^2+^ response of S170A (**P < 0.01). Data are shown as fold increase over the basal level of IP1 upon incubation in ligand buffer and are means ± S.E.M. of three independent experiments performed in triplicates.

In order to study the negative modulation of CaSR activity, Ca^2+^ was tested in the presence of 0.2 μM NPS 2143, 1 μM NPS 2143 and 5 μM NPS 2143, respectively. The S170A:F801A heterodimer that contains allosteric sites in both subunits displayed concentration-dependent inhibition by NPS 2143, in which full inhibition was observed at the highest tested NPS 2143 concentration (Fig. 6a). As expected, the activity of the S170A/E837A:F801A/E837A heterodimer was not subjected to inhibition by NPS 2143 (Fig. 6b), as the allosteric site in both subunits was impaired by mutation. The S170A:F801A/E837A dimer contains a functional allosteric site in the subunit responsible for G protein coupling and displayed concentration-dependent inhibition with full inhibition at 5 μM NPS 2143 (Fig. 6c), while NPS 2143 triggered only 30% inhibition of the Ca^2+^-induced max response of the S170A/E837A: F801A heterodimer that contains an allosteric site in the subunit unable to couple G proteins (Fig. 6d). Consequently, full inhibition of a dimer with only one NAM binding site was not observed when the adjacent subunit allowed G protein coupling, whereas the activity of the S170A:F801A/E837A dimer was fully prevented due to NAM binding in the S170A subunit and mutational impairment of G protein coupling in the F801A/E837A subunit. In accordance with results from the PAM testing, a small, but significant, increase in IP_1_ production was observed for the S170A/E837A mutant upon stimulation with 23 mM Ca^2+^, which was not subjected to inhibition by NPS 2143 due to the E837A mutation (Fig. 6e). No effect of Ca^2+^ or NPS 2143 was observed when testing S170A, F801A and F801A/E837A, respectively, and neither of the mutant homodimers thus had any influence on the responses measured for the heterodimers (Fig. 6e).

## Discussion

In the current study, we have shown that one allosteric site per CaSR dimer was sufficient for obtaining the modulatory effect of the PAM NPS R-568, while prevention of activation in both 7TM domains were required for achieving full inhibition by the NAM NPS 2143.

In order to investigate the allosteric modulation in CaSR, we needed an assay that allowed for measurements on a dimer comprised of well-defined subunits each carrying specific mutations. For that purpose, we applied a *trans*-activation system, in which only the heterodimer comprised of a mutant preventing agonist activation and a mutant preventing G protein coupling was functionally active. This *trans*-activation signaling mechanism has previously been reported for CaSR^33^. In the present study, the S170A and F801A mutants were chosen for setting up the *trans*-activation system, as F801A was non-functional at concentrations up to 45 mM Ca^2+^, while S170A demonstrated very limited activity at this concentration. S170 is a highly conserved residue located in the orthosteric binding site of class C receptors^26^,^36^,^45^,^46^, and F801 is found in the ICL3 in which it has proven crucial for G protein coupling in the mGluRs, CaSR and GABA_B_ receptors^27^,^38^,^39^. The specific S170A:F801A heterodimer displayed a recovery of receptor activity with a Ca^2+^-mediated response constituting 30% of the WT response. CaSR thus only requires one subunit for agonist binding and one subunit for G protein coupling, however the response was less efficacious than for the WT receptor. This is consistent with previously reported *trans*-activation responses, which also demonstrated a considerable decrease in max response^31^,^32^,^33^. For the mGluRs, it has been shown that agonist binding in both ATDs in the dimer was required for achieving the full receptor response^19^,^32^ and likewise, full activation was only obtained in dimers having both 7TM domains available for G protein coupling^29^. This would explain the observed decrease in efficacy for the *trans*-activation response in CaSR, as the heterodimer contains only one functional ATD and one subunit that allows G protein coupling. Furthermore, non-functional homodimers of each mutant was also formed upon the co-expression, and the functional proportion of receptors was thus smaller than for the WT receptor. In conclusion, we have confirmed that CaSR can signal via *trans*-activation, which supports that signal transduction from the ATDs to the 7TM domains depends on intersubunit rearrangements rather than intrasubunit movements in accordance with studies conducted on the mGluRs^31^.

By applying the *trans*-activation assay, it was possible to test CaSR dimers containing allosteric sites in one or both of the subunits. The responses of a heterodimer containing an allosteric site in both subunits (S170A:F801A) and a heterodimer with only one allosteric site (S170A:F801A/E837A) were both subjected to potentiation upon addition of NPS R-568, and our findings thus proved that one allosteric site was sufficient for obtaining potentiation of the agonist response in CaSR. Importantly, this potentiation was only achieved when the PAM was bound to the subunit in which G protein coupling occurred. If the allosteric site was located in a subunit with impaired G protein coupling, a small NPS R-568-mediated inhibition was instead observed. These findings are in perfect correlation with results obtained for an mGluR5/mGluR1 dimer, in which an mGluR5 PAM fully enhanced the agonist response in spite of the dimer having only one mGluR5 PAM site, while an mGluR1 PAM acted as a non-competitive antagonist, when G protein coupling was prevented in the mGluR1 subunit^30^.

Although the applied S170A mutant was virtually inactive, substantial NPS R-568-mediated potentiation was observed, hence suggesting that the mutation did not completely eliminate agonist activation in CaSR, although the potency of Ca^2+^ was greatly reduced. It is well established that agonist binding in CaSR is highly cooperative with a Hill coefficient of 3–4^47^, thus strongly indicating that numerous agonist binding sites are found in this receptor. At least five putative Ca^2+^ sites were identified in CaSR by use of homology modeling^47^,^48^, while recent crystal structures of the ATD provided further evidence to the existence of multiple divalent cation sites^13^,^14^, although the precise location differed between studies. Additionally, it has been shown that agonist stimulation of CaSR triggers an increase in the level of receptors at the cell surface, which also contributes to the cooperativity of the Ca^2+^-mediated response of CaSR^49^. In the present study, the low potent activity of the S170A construct seems likely to arise from the additional agonist binding sites in the receptor. While the NPS R-568-mediated activity of S170A was likely to contribute to the responses measured upon co-expression of S170A and F801A or S170A and F801A/E837A, the S170A homodimers alone was not sufficient to trigger the observed potentiation, hence demonstrating that the S170A:F801A and S170A:F801A/E837A heterodimers must also be subjected to potentiation. Nevertheless, it is difficult to evaluate the exact contributions from S170A against the contributions from the relevant heterodimer, and is has therefore not been possible to asses the extent of potentiation for the heterodimers in the current study. This limitation should be kept in mind when interpreting the results from testing PAMs at the heterodimers.

Negative modulation of receptor activity was likewise studied by using CaSR dimers carrying mutations in one or both of the allosteric sites. Results showed that the activity of the S170A:F801A/E837A dimer with a single allosteric site was subjected to full inhibition by 5 μM NPS 2143, and the NAM thus seemed able to completely inhibit the activity of CaSR when binding in only one subunit. Noticeably, neither of the subunits in the S170A:F801A/E837A dimer was however able to trigger intracellular signaling, as the F801A mutation was impairing G protein coupling in the adjacent subunit. The S170A/E837A:F801A dimer also contained only one allosteric site, however NPS 2143 triggered only 30% inhibition of the max response of this receptor dimer, as the S170A/E837A subunit was still available for activation and G protein coupling. In conclusion, only a dimer in which activation and/or G protein coupling were prevented in both 7TM domains demonstrated full inhibition of CaSR activity. These results also correlate well with studies conducted using the mGluRs, for which it was shown that full inhibition required binding of antagonist in both subunits^29^.

According to the model of receptor activation that has been proposed for the mGluRs, only one 7TM domain in the dimer is activated at a time^29^,^30^. The 7TM domain alternates between active and inactive conformations, of which PAM favors the active conformation that allows for G protein coupling while NAM stabilizes the 7TM domain in an inactive conformation. In the present study, potentiation was observed for dimers containing only one allosteric site, which suggests that only one 7TM domain in CaSR is stabilized in an active conformation. This model also provides an explanation to the NPS R-568-mediated inhibition of activity for the S170A/E837A:F801A dimer, as PAM binding in F801A favored activation of this subunit in spite of the impairment of G protein coupling. Favoring the active conformation of one subunit would simultaneously prevent activation of the adjacent subunit, and signaling in the S170A/E837A subunit was thereby inhibited by NPS R-568. Our results from testing negative modulation in CaSR further supports this activation mechanism as inhibition of activity in both subunits would be required for fully preventing receptor signaling if a single 7TM domain is activated at a time. Accordingly, full inhibition of CaSR was observed only when one subunit was inactivated by NAM whilst G protein coupling was impaired in the other subunit. On the contrary, the S170A/E837A:F801A heterodimer retained 70% activity as the S170A/E837A subunit was still available for G protein coupling.

In conclusion, we have shown that one allosteric site per dimer was sufficient for obtaining potentiation of the agonist-mediated activity of CaSR, while prevention of activation in both 7TM domains was required for full inhibition of the functional response. These findings correlate with the current model for mGluR activation, in which only one 7TM domain is activated at a time, and our study thus suggests that the activation mechanism is shared among the class C receptors. This asymmetry in the activation of the 7TM domains is however puzzling, as dimerization has proven crucial for the functional coupling between the ATDs and the 7TM domains, and more studies are therefore required in order to fully understand the molecular basis for activation and modulation in the 7TM domains of class C receptors. Such knowledge would be highly beneficial for the development of much needed novel therapeutics targeting CaSR.

## Methods

### Materials

Dulbecco’s modified eagle medium (DMEM), 10.000 units/mL penicillin and 10.000 μg/mL streptomycin mixture (P/S), dialyzed fetal bovine serum (dFBS), Opti-MEM, Dulbecco’s Phosphate Buffered Saline (DPBS), Hank’s Balanced Salt Solution (HBSS) and Lipofectamine 2000 were all purchased from Thermo Fisher Scientific (Waltham, MA, USA). NPS R-568 hydrochloride was purchased from Tocris Bioscience (Bristol, UK), while NPS 2143 hydrochloride was synthesized as previously published^50^ and kindly provided by Dr. Daniel Sejer Pedersen, University of Copenhagen, DK. All other chemicals were purchased from Sigma-Aldrich (St. Louis, MO, USA) unless otherwise stated.

### Constructs

The previously described myc-tagged rat GPRC6A-pEGFPN1 construct, which contains the mGluR5 signal peptide upstream of the myc tag^51^, was used as template for generating the CaSR constructs used in the present study. myc-tagged CaSR was generated by replacing the rat GPRC6A sequence with the rat CaSR sequence by use of the restriction sites MluI and NotI. A sequence comprised of the mGluR5 signal peptide and an HA tag was synthesized by Genscript (Piscataway, NJ, USA) and inserted in myc-CaSR-pEGFPN1 instead of the mGluR5 signal peptide and the myc tag by use of XhoI and MluI thus generating HA-CaSR. All mutants were generated by Genscript (Piscataway, NJ, USA) using the HA-CaSR-pEGFPN1 construct as template and mutations were verified by sequencing. myc-tagged mutants were generated by exchanging the mGluR5 signal peptide and HA-tag sequence with the mGluR5 signal peptide and myc-tag sequence by using the XhoI and MluI restriction sites.

### Cell culturing and transfection

Human embryonic kidney (HEK) 293T cells were cultured in DMEM supplemented with 10% dFBS and 1% P/S at 37 °C and 5% CO_2_. 48 h prior to assay, transient transfection was performed using Lipofectamine 2000 as the transfection reagent. White opaque 96-well culture plates (PerkinElmer, Waltham, MA, USA) were used for the ELISA and the TR-FRET dimerization assay, while clear tissue culture treated 96-well plates (Corning, Corning, NY, USA) were used for the IP-One assay. Both plate types were coated with poly-d-lysine before addition of cells and transfection mix. 0.25 μl/well of Lipofectamine 2000 were diluted in 25 μl/well of Opti-MEM and incubated at room temperature (RT) for 5 min. 0.03 μg/well of DNA was diluted in 25 μl/well of Opti-MEM, and subsequently mixed with the Lipofectamine 2000 dilution and incubated 20 min at RT before addition to the plates. Cell suspensions of 170.000 HEK293T cells/mL were prepared in complete growth medium and 100 μl/well (17.000 cells/well) was added to the plates, which were incubated for 48 h at 37 °C and 5% CO_2_ before performing assays.

### ELISA

For assessment of surface and total expression of WT and mutant CaSR constructs, ELISA was performed as previously described^44^. In brief, cells were fixated using 4% paraformaldehyde in DPBS for 5 min at RT. For measurements of total expression, cells were permeabilized using 0.1% Triton-X in DPBS. All wells were incubated in blocking solution (ddH_2_O with 3% skim milk, 1 mM Ca^2+^, 50 mM Trizma hydrochloride solution, pH 7.4) for 30 min after which primary antibody incubation for 45 min at RT was performed by addition of either anti-HA antibody (Nordic BioSite, Täby, Sweden) or anti-myc antibody (Thermo Fisher Scientific, Waltham, MA, USA) diluted 1:1000 in blocking solution. Subsequently, horseradish peroxidase conjugated anti-mouse antibody (GE Healthcare Biosciences, Pittsburgh, PA, USA) diluted 1:1500 in blocking solution was added and plates were incubated for 45 min at RT. Receptor levels were detected by addition of 80 μl/well of DPBS supplemented with 1 mM CaCl_2_ and 10 μl/well of SuperSignal ELISA Femto Substrate (Thermo Fisher Scientific, Waltham, MA, USA). Chemiluminescence was measured on an EnSpire plate reader (PerkinElmer, Waltham, MA, USA) and normalized to the level of WT CaSR.

### Functional IP-One assay

Functional activity was measured using the IP-One assay, which is an IP_1_ accumulation assay^52^. For agonist testing, ligands were prepared in final concentrations in ligand buffer (HBSS, 20 mM HEPES, pH 7.4 supplemented with 20 mM LiCl). Cells were washed once with 100 μl/well of assay buffer (HBSS, 20 mM HEPES, pH 7.4) and 50 μl/well of agonist solution was added after which plates were incubated at 37 °C for 30 min. Subsequently, ligand solutions were removed and cells were washed with 100 μl/well of assay buffer. Cell lysis was performed by addition of 30 μl/well of IP-One Conjugate & Lysis Buffer (Cisbio Bioassays, Codolet, France) and plates were incubated at RT for 30 min. Afterwards, 30 μl/well of assay buffer was added to dilute the cell lysate. 10 μl/well of cell lysate was transferred from the 96-well plate to a 384-well Optiplate and 10 μl/well of detection solution (Assay buffer + 2.5% of anti-IP_1_ antibody cryptate Terbium conjugate + 2.5% IP_1_-d2 conjugate (Cisbio Bioassays, Codolet, France)) was also added. The 384-well plate was incubated for 1 h at RT in dark before being measured on an EnVision plate reader (PerkinElmer, Waltham, MA, USA). Upon excitation at 340 nm, emission at 615 nm and 665 nm was measured, and the FRET ratio (665 nm/615 nm) was converted to IP_1_ concentrations by interpolating values from an IP_1_ standard curve generated from a provided IP_1_ calibrator (Cisbio Bioassays, Codolet, France). When testing allosteric modulators, cells were pre-incubated for 30 min at 37 °C with 50 μl/well of ligand buffer supplemented with allosteric modulator and a final concentration of 1% DMSO. Afterwards, the buffer solution was removed and 50 μl/well of agonist solution in ligand buffer supplemented with allosteric modulator and a final concentration of 1% DMSO was added. Plates were incubated for 30 min at 37 °C followed by one wash in 100 μl/well of assay buffer. Cell lysis and detection was performed as described above.

### TR-FRET dimerization assay

Dimerization of a HA-tagged receptor construct and a myc-tagged receptor construct was assessed by a TR-FRET-based assay as previously described^44^. In brief, cells were washed once in 100 μl/well of 4 °C assay buffer (HBSS, 20 mM HEPES, 0.5 mM CaCl_2_, 0.5 mM MgCl_2_, 5 mg/mL bovine serum albumin, pH 7.4) before addition of the fluorophore-conjugated antibodies. Tb^3+^ conjugated anti-HA antibody (Cisbio Bioassays, Codolet, France) was used as the donor fluorophore and was diluted in 4 °C assay buffer to a final concentration of 1 nM, while d2 conjugated anti-myc antibody (Cisbio Bioassays, Codolet, France) was used as the acceptor fluorophore and was diluted in 4 °C assay buffer to a final concentration of 50 nM. 25 μl/well of each fluorophore was added to the plate, which was incubated for 24 h at 4 °C. Cells were washed four times with 100 μl/well of 4 °C assay buffer before being measured on an EnVision plate reader (PerkinElmer, Waltham, MA, USA). Upon excitation at 340 nm, emission at 615 nm and 665 nm were measured, and the FRET ratio (665 nm/615 nm) was calculated for each condition and normalized to the FRET ratio of pEGFPN1.

### Data analysis

Data were analyzed using GraphPad Prism version 6 (GraphPad Software, San Diego, CA) and normalized as indicated in figure legends.

## Additional Information

**How to cite this article**: Jacobsen, S. E. *et al*. Investigating the molecular mechanism of positive and negative allosteric modulators in the calcium-sensing receptor dimer. *Sci. Rep.* **7**, 46355; doi: 10.1038/srep46355 (2017).

**Publisher's note:** Springer Nature remains neutral with regard to jurisdictional claims in published maps and institutional affiliations.

## Figures and Tables

**Table 1:** Functional parameters of WT CaSR and constructs with mutations in either the ATD or ICL3 region.

	EC_50_ (mM)	pEC_50_ ± S.E.M.	Max ± S.E.M. (%)^a^	Hill coefficient ± S.E.M.
HA-CaSR WT	3.77	2.42 ± 0.01	100	3.62 ± 0.48
HA-S147A	9.07	2.04 ± 0.01	150 ± 17	3.50 ± 0.26
HA-S170A	>45	N.D.	17 ± 0.7	N.D.
HA-D190A	17.8	1.75 ± 0.01	110 ± 11	3.77 ± 0.39
HA-E297A	7.36	2.31 ± 0.02	130 ± 25	3.01 ± 0.07
HA-S147A/S170A	9.04	2.04 ± 0.02	130 ± 28	3.00 ± 0.07
HA-S170A/D190A	31.8	1.50 ± 0.04	49 ± 2.5	4.05 ± 0.43
HA-L797A	11.7	1.94 ± 0.04	25 ± 3.5	3.22 ± 0.71
HA-F801A	>30	N.D.	5.8 ± 0.4	N.D.

Ca^2+^-mediated activation of the G_q_ signaling pathway was measured using the IP-One assay, and data are means ± S.E.M. of three independent experiments performed in triplicates.

^a^The maximum response for each mutant is normalized to the maximum response of WT CaSR.

EC_50_: the concentration of Ca^2+^ that is required for eliciting 50% of the maximum response. pEC_50_ = −log(EC_50_).

N.D.: not determined due to a very low activity at the highest tested Ca^2+^ concentration.

**Table 2:** Functional parameters of WT CaSR and the *trans*-activation response arising from the S170A:F801A heterodimer.

	EC_50_ (mM)	pEC_50_ ± S.E.M.	Max ± S.E.M. (%)^a^	Hill coefficient ± S.E.M.
HA-CaSR WT + myc CaSR-WT	2.98	2.53 ± 0.02	100	4.24 ± 0.19
HA-S170A + myc-F801A	12.7	1.90 ± 0.03	30 ± 1.9	2.69 ± 0.19

Ca^2+^-mediated activation of the G_q_ signaling pathway was measured using the IP-One assay, and data are means ± S.E.M. of three independent experiments performed in triplicates.

^a^The maximum *trans*-activation response is normalized to the maximum response of WT CaSR.

EC_50_: the concentration of Ca^2+^ that is required for eliciting 50% of the maximum response. pEC_50_ = −log(EC_50_).

**Table 3:** Functional parameters of WT CaSR and constructs with mutations in the allosteric site.

	Ca^2+^					
	EC_50_ (mM)^a^	pEC_50_ ± S.E.M.	Max ± S.E.M. (%)^b^			
HA-CaSR WT	3.21	2.49 ± 0.01	≈100			
HA-F684A	6.60 (***)	2.18 ± 0.01	97 ± 3.4			
HA-F688A	6.11 (***)	2.21 ± 0.01	86 ± 3.1			
HA-F821A	2.35 (***)	2.63 ± 0.02	87 ± 4.3			
HA-E837A	3.40 (ns)	2.47 ± 0.01	87 ± 16			
HA-I841A	2.59 (**)	2.59 ± 0.01	81 ± 14			
	Ca^2+^ with 1 μM NPS R-568			Ca^2+^ with 5 μM NPS 2143		
	EC_50_ (mM)^c^	pEC_50_ ± S.E.M.	Max ± S.E.M. (%)^d^	EC_50_ (mM)^c^	pEC_50_ ± S.E.M.	Max ± S.E.M. (%)^d^
HA-CaSR WT	1.28 (***)	2.89 ± 0.01	110 ± 11	8.40 (***)	2.08 ± 0.01	72 ± 5.6
HA-F684A	2.89 (***)	2.54 ± 0.02	99 ± 11	8.17 (***)	2.09 ± 0.004	70 ± 8.1
HA-F688A	2.86 (***)	2.54 ± 0.02	110 ± 9	8.31 (***)	2.08 ± 0.01	79 ± 6.9
HA-F821A	1.92 (ns)	2.72 ± 0.01	110 ± 14	5.72 (**)	2.25 ± 0.05	51 ± 8.0
HA-E837A	3.88 (ns)	2.41 ± 0.04	100 ± 5	3.46 (ns)	2.46 ± 0.02	91 ± 5.7
HA-I841A	3.68 (ns)	2.54 ± 0.05	99 ± 18	5.29 (*)	2.28 ± 0.03	97 ± 7.6

Activation of the G_q_ signaling pathway was measured using the IP-One assay, in which the endogenous agonist Ca^2+^ was tested in the presence and absence of 1 μM NPS R-568 and 5 μM NPS 2143, respectively. Data are means ± S.E.M. of three independent experiments performed in triplicates.

^a^Statistical analysis was performed using one-way ANOVA followed by Dunnett’s post-test in which the EC_50_ of Ca^2+^ for each mutant was compared to the EC_50_ for WT CaSR (ns, *P* > 0.05, **P < 0.01, ***P < 0.001).

^b^The maximum response for each mutant when stimulating with Ca^2+^ alone was normalized to the maximum response of WT CaSR.

^c^Statistical analysis was performed using one-way ANOVA followed by Dunnett’s post-test in which the EC_50_ of Ca^2+^ in the presence of PAM or NAM for each construct was compared to the EC_50_ for Ca^2+^ alone for that construct (ns, *P* > 0.05, *P < 0.05, **P < 0.01, ***P < 0.001).

^d^The maximum response when stimulating with Ca^2+^ in the presence of NPS R-568 or NPS 2143 is normalized to the maximum response when stimulating with Ca^2+^ alone for each construct.

EC_50_: the concentration of Ca^2+^ that is required for eliciting 50% of the maximum response. pEC_50_ = −log(EC_50_).
